# Remarkable Case of Mental Alienation

**Published:** 1846-06

**Authors:** G. T. Wragg


*Remarkable Case of Mental Alienation.* By G. T. Wragg, M. D. —Joe, a young negro of about 20 years of age, possessing an average degree of intelligence, and having enjoyed good health up to the time when he was attacked with the illness which threw him into the remarkable condition in which I found him, resided on a plantation in the neighborhood of Charleston. His occupations were such as are common to persons in his situation; laboring in the cultivation of the soil. He was taken ill about the latter part of July, 1837, probably with fever of a bilious type. For about a fortnight he remained on the plantation, receiving such attention as the neighborhood afforded. It does not appear that he suffered from neglect, but it seemed evident that his case had not been judiciously treated. A report of his death reached his master, who resided in Charleston, and was the first intimation that the boy was really seriously ill. This report caused a careful inquiry to be made into his real condition when it was found that although he was not dead, yet his case was of so serious a character as to call for most careful attention. He was therefore brought to town. I saw him for the first time on Tuesday, 8th August, 1837, and found him laboring under violent delirium and a great deal of muscular irritability. I could obtain no satisfactory account of the manner in which the attack commenced, or of the nature and progress of the disease; except that he had had fever. His imagination, a faculty which with him, had doubtless never, in his hours of health, been called into action, was awakened. He became impressed with the idea that he was dead. This, no doubt, originated from the report of his death, which had been current, and which had probably been spoken of in his presence.

Upon a mind laboring under so much excitement, and in which the exaggerations of timid and ignorant friends could find no counterpoise in the wholesome restraints of education, such an idea was well calculated to produce a deep impression, and accordingly the patient was hurried away in the most extravagant language and conduct. From this false ground he drew inferences perfectly secutive, and which failed to be rational, only because they started from unsound premises. He said, that being dead, his flesh would soon begin to rot and drop from his bones; remonstrated at being kept so long unburied; earnestly demanded that his grave clothes should be prepared and put upon him, and that he be laid out in the usual form. He looked anxiously for the company to assemble which was to follow his body to the grave, and would chaunt in touching language a final adieu to his mother. The tune he selected was solemn, such as he was used to hear under similar circumstances. He would pick and pull at his flesh, while he called on the bystanders to look at him closely, and satisfy themselves that he really was dead.

In the midst of these interesting and deeply touching scenes, he would sometimes burst suddenly into a fit of hearty laughter, and then as suddenly, as if rebuked by his conscience for the indecent levity of such conduct in one who was already an inhabitant of another world, he would check his mirth. And then, his countenance would be marked in every feature with an expression of the deepest solemnity, he would address himself earnestly to some object in the room, as though he were in the presence of the Almighty, and deeply awed by his majesty ; he would then in the most earnest and appropriate language, give expression to his feelings of reverence for the Great Being in whose presence he stood ; and with the attitude and accent of prayer, acknowledge his power to save or to destroy. Fully impressed with the idea that he was indeed dead, and a dweller amongst immortal spirits in that world which his religious instruction had taught him to believe would receive his soul, when death had released it from its fleshy tenement; his countenance and his every action took a serious, a sublime expression from the thought, and his whole deportment was such as could not fail to touch, and awe all who saw him. He would remain for some time enveloped in this rhapsody. He heard nothing of what passed near him, and saw only the majestic creation of his imagination, and lived only in regions which his mental infirmity had painted, till they seemed to him those of another and brighter world.

Gradually, after a day or two, his delirium took a character of gayety. His countenance wore a pleasant smile, and a vein of humor marked his conversation. But, if opposed, he would resist forcibly ; making powerful muscular efforts, and once he inflicted a severe blow on one of the bystanders. He would sing a tune with perfect accuracy, adapting to it, as he proceeded, words suggested by what was passing around him. When questioned, he would chaunt his answer with perfect correctness, thus conveying all the required information concerning his feelings, his wishes and his thoughts. His gestures were easy and appropriate, nor could he be restrained from making them, by any mechanical opposition placed in his way ; for there was a rigid and unyielding energy in his muscular contractions, that overpowered all resistance, like the delirious and convulsive movements of a patient laboring under phrenitis. Restraint made him violent; but if he succeeded in releasing himself, or the restraint was removed, upon the instant a mild and gentle smile threw its bland expression over his face, and he became obedient to a single word, if uttered in a gentle tone. He knew his mother, and always spoke to her with kindness.

This musical mania continued for two days. About the third day, he ceased singing, and the most remarkable peculiarity that his delirium had yet assumed now presented itself. He spoke in rhyme! As he had before made all his answers to the questions put to him, and his voluntary remarks, in a measured and musical tone, so did he now communicate his thoughts in well selected rhymes. He would sometimes rhyme repeatedly on the same word. Again, his transitions wouldbe rapidly made from one sound to another, of an entirely different kind. At all times the words were so selected as to make the most perfect rhyme. And he displayed a degree of ingenuity in collecting and bringing into use, in the most apposite manner, a large number of similarly sounding words, which would have appeared astonishing even in one who had been rendered familiar, by education and habit, with language in all its perfection. But it must be remembered that this individual was a slave, perfectly uneducated, and showing no farther knowledge of language than was sufficient for expressing his few and simple wants. Negroes have some quickness in catching musical sounds, and in repeating simple tunes by air; hence, I was not surprised to hear him sing; though the quickness with which he adapted his answers and remarks to the tune he was singing, was indeed remarkable. But the ease with which he rhymed was truly astonishing.

The medical history and treatment of the case were as follows:

The patient had been long suffering for want of rest, and for several nights continued to be sleepless. He had well marked exacerbation towards night, when his pulse, which throughout the day would continue nearly at a healthy standard, became quick, small and irritable. During the exacerbations, he paid no attention to what was said to him, unless spoken to earnestly. He would then listen attentively, and would readily promise obedience to any directions; and he always kept his word.

After repeated bleeding, both general and local, blistering, purging, hot pediluvia with mustard, and other means of depletion and derivation, his madness became more calm, but he never said any thing rational; only making in various ways a few half intelligible complaints of the blister, which had been put on his head. His rest returned to him. He would sleep well at night, and frequently had refreshing naps through the day. His appetite became good, so that he eat heartily and with relish.

On the 17th of August, nine days after I first saw him, when the depletory revulsive measures mentioned, had removed all symptoms of excitement except the delirium, a seaton was put in the back of the neck. He was very much alarmed at the idea of the operation, and the sight of the knife and other preparations caused him to shudder, and it was evident he suffered as much mental as bodily pain. But when assured that the operation would benefit him and that it was only for his own good, he became calm, and expressed himself thankful.

The seaton remained till Saturday, the 14th October, forty-eight days from the time of its insertion. The delirium abated evidently and steadily, from the time that suppuration was established. He soon began to walk about the room, then in the street near his residence, and gradually extending his promenade, came to see me at my office and report on his condition. When the seaton was removed, his intellect was perfectly clear, and his physical health completely restored. No inconvenience resulted from the drying up of the suppuration, and he returned to his occupation in perfectly restored health.—*Southern Journal of Medicine and Pharmacy.*

